# Supplementary material for: Clinical, laboratory, and hospital factors associated with preoperative complications in Peruvian older adults with hip fracture
Source: PLoS One. 2024 Nov 4;19(11):e0313089. doi: 10.1371/journal.pone.0313089 (PMC11534202; doi:10.1371/journal.pone.0313089)
Supplement: Attachment — Submitted filename: Response to Editor.pdf [file pone.0313089.s002.pdf]

Dear editor, it was modified according to your recommendations.

(1) Please upload an English translation of the original ethics approval document as a Supporting Information file. Please note that if this document is not included when your manuscript is resubmitted, it may be rejected.
Our response: The ethics document was translated. It was uploaded to the platform. Thank you very much.

(2) Please report in the Methods section the dates when data were accessed for research purposes. Please note that if this information is not included when your manuscript is resubmitted, it may be rejected.
Our response:
We have added the recommendations on the dates on which the data was accessed in the process and methodology techniques section.

Data collection started on January 6, 2022, and continued until March 28, 2022, using the data collection form with the patient record book of the orthogeriatric unit.

We uploaded the database to a public repository with encrypted data for submission purposes and to adhere to PLOS ONE author guidelines.

https://doi.org/10.6084/m9.figshare.26507296.v1

We have resolved the observations. If you have any questions, we will be delighted to answer them.
Sincerely,

Mario J. Valladares-Garrido

Universidad Continental, Lima 15046, Peru; mvalladares@continental.edu.pe
