# Supplementary material for: Clinical, laboratory, and hospital factors associated with preoperative complications in Peruvian older adults with hip fracture
Source: PLoS One. 2024 Nov 4;19(11):e0313089. doi: 10.1371/journal.pone.0313089 (PMC11534202; doi:10.1371/journal.pone.0313089)
Supplement: Attachment — Submitted filename: Response to Editor.pdf [file pone.0313089.s003.pdf]

Dear **Editor**,

Thank you very much for reviewing our article, **"Clinical, laboratory, and hospital factors associated with preoperative complications in Peruvian older adults with hip fracture".** Your suggestions and comments will be addressed below. Thank you for your valuable time and excellent review.

Editor's comments

1. Das Link inaccessible
Please note that your Data Availability Statement is currently missing [the repository name and/or the DOI/accession number of each dataset OR a direct link to access each database]. If your manuscript is accepted for publication, you will be asked to provide these details on a very short timeline. We therefore suggest that you provide this information now, though we will not hold up the peer review process if you are unable.

Our response:

The database has been uploaded to the figshare website (http://doi.org/10.6084/m9.figshare.26507296.v1). The file can be downloaded.

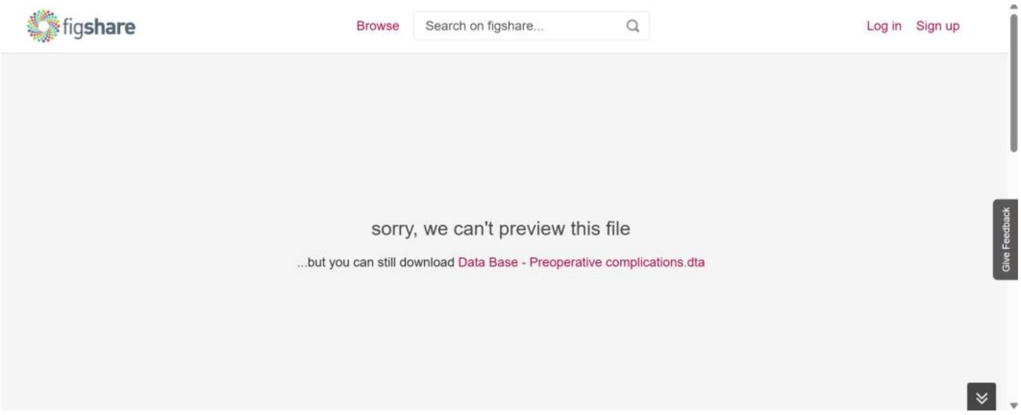

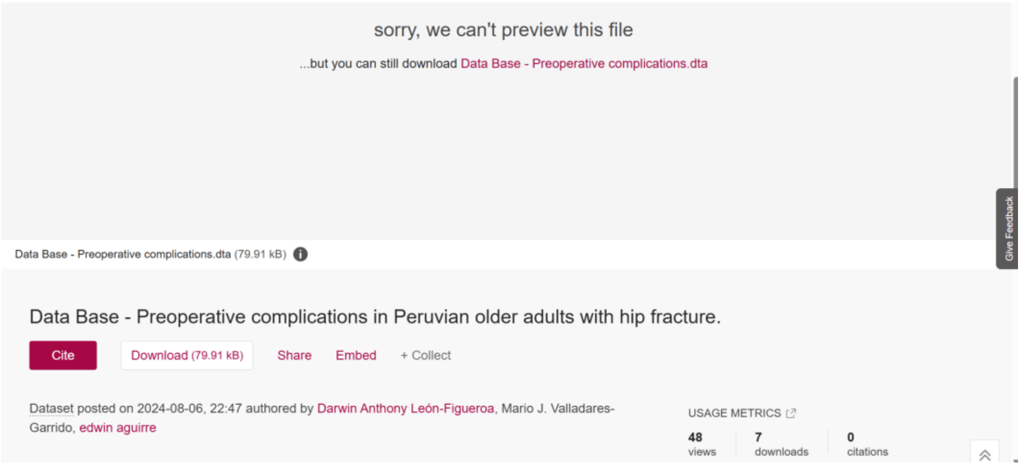

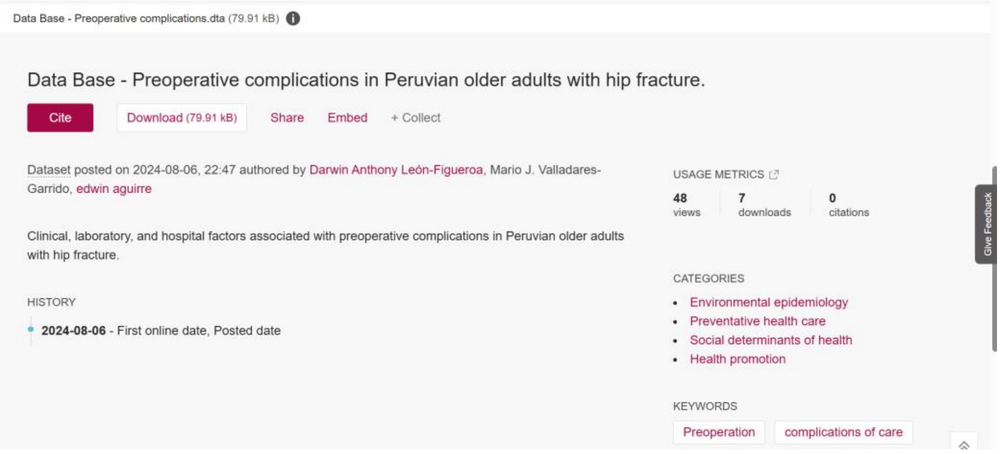
Data Base - Preoperative complications.dta (79.91 kB)

Data Base - Preoperative complications in Peruvian older adults with hip fracture.

Cite | Download (79.91 kB) | Share | Embed | + Collect

Dataset posted on 2024-08-06, 22:47 authored by Darwin Anthony León-Figueroa, Mario J. Valladares-Garrido, edwin aguirre

Clinical, laboratory, and hospital factors associated with preoperative complications in Peruvian older adults with hip fracture.

HISTORY

- **2024-08-06** - First online date, Posted date

USAGE METRICS

48 views | 7 downloads | 0 citations

CATEGORIES

- Environmental epidemiology
- Preventative health care
- Social determinants of health
- Health promotion

KEYWORDS

Preoperation | complications of care

Give Feedback

I confirm that the file is available for download on the platform and at the indicated link. However, it is not possible to preview the file, as it is in .dta format, specific to the Stata program. For this reason, it is necessary to download it to access its contents.

Sincerely,

Mario J. Valladares-Garrido

Universidad Continental, Lima 15046, Peru; mvalladares@continental.edu.pe
